# Migration-Related Stressors and Their Effect on the Severity Level and Symptom Pattern of Depression among Vietnamese in Germany

**DOI:** 10.1155/2017/8930432

**Published:** 2017-08-22

**Authors:** Simon Wolf, Eric Hahn, Michael Dettling, Main Huong Nguyen, Katja Wingenfeld, Markus Stingl, Bernd Hanewald, Thi Minh Tam Ta

**Affiliations:** ^1^Charité-Universitätsmedizin Berlin (Corporate Member of Freie Universität Berlin, Humboldt-Universität zu Berlin, and Berlin Institute of Health), Department of Psychiatry and Psychotherapy, Campus Benjamin Franklin, Berlin, Germany; ^2^Department of Psychiatry and Psychotherapy, Universitätsklinikum Gießen, Gießen, Germany

## Abstract

**Objectives:**

Vietnamese in Germany represent a scarcely researched and vulnerable group for mental health problems, especially under exposure to migration-related stressors (MRS). This study analyzes the effect of those MRS on the severity level and symptom pattern of depression.

**Design:**

We analyzed the data of 137 depressed Vietnamese patients utilizing Germany's first Vietnamese psychiatric outpatient clinic. Hierarchical linear regression models were applied to investigate how the quantity of MRS influenced (1) the overall severity of self-reported depression symptoms; (2) the cognitive, affective, and somatic BDI-II subscale; and (3) the single BDI-II items of these subscales.

**Results:**

A greater number of MRS were related to a higher severity level of depression in general, as well as to a higher level on the cognitive depression subscale in particular. The BDI-II single items* pessimism*,* past failure*,* guilt feelings*,* punishment feelings*, and* suicidal thoughts* were particularly associated with a higher quantity of perceived MRS.

**Conclusion:**

Among depressed Vietnamese migrants in Germany, a higher number of reported MRS were associated with higher overall depression severity. Within the domains of depression, particularly the cognitive domain was linked to perceived MRS. The association between MRS and suicidal thoughts is clinically highly relevant.

## 1. Introduction

Most migration processes may be conceptualized as a series of mainly stressful life events, each with the cumulative capacity to increase the risk for a broad range of mental health problems. While from a global perspective mental health issues are widely spread among migrants worldwide [1] findings on the epidemiology regarding specific mental disorders still vary with respect to different migration-samples and migration-contexts. This inconsistency also holds true to the incidence and prevalence rates of mood disorders among migrants. While a few studies have reported either similar [2] or even lower [3] depression rates compared to the mainstream population, the majority of these studies have found increased prevalence rates [4]. In one recent European study, for example, the prevalence rates of elderly Moroccan (33.6%) and Turkish (61.5%) migrants turned out to be significantly higher than in a native Dutch sample (14.5%) [5]. Additionally, many of these studies do not merely report increased depression rates but also a higher severity level and prolonged course of depression with less mental health care utilization in migrants [6].

Regarding Vietnamese in Germany, although being the country's largest Southeast Asian migrant population, research on their mental health condition still is extremely rare. Among the very few existing studies a survey of 88 Vietnamese migrants in the city of Leipzig identified higher anxiety and depression scores as well as a lower utilization of medical and psychosocial institutions than in the German control sample [7]. Moreover, perceived discrimination was shown to be a powerful migration-related stressor (MRS, defined as non-life-threatening but highly stressful events caused by the migration process), having a substantial influence on the level of depression and anxiety [7]. The latter finding underscores the importance of further clarifying the mechanism of how mental health problems, such as anxiety or mood disorders, develop under the influence of acculturative stressors.

This possible accumulation of psychological distress among Vietnamese migrants may be further aggravated by their delayed mental health care utilization. These findings have been reported in numerous studies worldwide [8] as well as in a German cross-sectional study contrasting Russian and Vietnamese outpatients in Berlin [9, 10]. Several reasons that may contribute to this underutilization have been documented: (a) One of them is difficulties of access for migrants in general due to language problems and the lack of health insurance [11]. (b) A tendency in Asian migrants to underreport psychological complaints together with an increased awareness of somatic symptoms in relation to a higher social acceptance of physical symptom expression may lead to utilizing general practitioners instead of psychiatrists [12]. (c) Most Asian and particular Vietnamese migrants may adhere to competitive traditional health beliefs and traditional or religious practices that differ from medical, psychiatric approaches [13]. (d) Vietnamese migrants that utilize the German mental health care system may express psychological distress in a different, culturally shaped way including expressions of a “social smile” that may lead to misdiagnosis when applying a non-culture-sensitive diagnostic perspective [14].

Thus, taking into account the fact that Vietnamese in Germany are highly undersupplied and at the same time repetitively exposed to acculturative stressors, the understanding of how this exposure contributes to the occurrence and course of common mental health problems, such as unipolar depression, might help to develop culture-adapted treatment options and to increase treatment adherence. Therefore, the current study investigates a clinical sample of clinically depressed Vietnamese migrants in Germany: (a) if and how the quantity of experienced migration-related stressors relates to the general severity level of self-reported depressiveness; (b) to which extent the number of experienced MRS has an effect on the affective, cognitive, and somatic dimension of depression; (c) which single depression symptoms within these dimensions are particularly associated with an increased number of MRS.

## 2. Methods

### 2.1. Procedure

The data was collected from March 2012 through February 2016 within the specialized psychiatric outpatient unit for Vietnamese migrants located at the Clinic for Psychiatry and Psychotherapy, Charité-Universitätsmedizin Berlin, Germany. All patients were initially screened with the Mini International Neuropsychiatric Interview (M.I.N.I. 5.0 [15]) by trained bilingual (i.e., German and Vietnamese) psychiatrists. Subsequently, all patients with a clinical relevant mood disorder answered the second version of the Beck Depression Inventory (BDI-II [16]), which had been pretranslated into written Vietnamese language using the technique of back-translation [17]. Afterwards, all patients received a questionnaire in the Vietnamese language about sociodemographic facts and potentially experienced migration-related stressors (MRS). The latter were assessed with an adapted version of 25 questions originally formulated by Lujic (2008) for the treatment evaluation of Turkish inpatients in Germany (see next section) [18].

Data used for this study was exclusively collected at patients' first admission before undergoing treatment. All subjects gave written informed consent before participating in this study. The study was approved by the ethics committee of the Charité-Universitätsmedizin-Berlin, Germany, and was conducted in line with the Declaration of Helsinki and its later amendments.

### 2.2. Instruments and Variables

#### 2.2.1. Depressive Symptoms

Depressive symptoms were assessed with Becks-Depression-Inventory II (BDI-II). The BDI-II, as a revision of the BDI, is a DSM-IV based self-report inventory for adolescents and adults, measuring the severity of depressive symptoms experienced over the last two weeks [16]. It contains 21 items with each of them being scored on a 4-point Likert scale (except for two items, i.e., change in sleeping pattern and change in appetite, containing seven response options). Individual item scores are summed up to provide a total score ranging from 0 to 63 with a higher value indicating greater symptom severity.

Since there is no validated Vietnamese version of the BDI-II published, we translated the German BDI-II version [19] into written Vietnamese language using the technique of back-translation [17], done by different bilingual translators.

For an analysis of how the quantity of experienced MRS relates to various facets of self-reported depressiveness, we applied the BDI-II symptom-structure model suggested by Buckley et al. (2001) [20]. This factor-analytic derived model clusters the 21 BDI-II items into three equivalent subgroups, labeled as the cognitive, affective, and somatic dimension of depression. Accordingly, we summed up (1) the items* sadness*,* pessimism*,* past failure*,* loss of pleasure*,* guilt feelings*,* punishment feelings*,* self-dislike*,* self-criticalness*,* suicidal thoughts, *and* worthlessness *for the BDI-COG-B subscale; (2) the items* loss of pleasure*,* crying*,* loss of interest, *and* indecisiveness *for the BDI-AFF-B subscale; (3) the items* agitation*,* loss of energy, change in sleeping pattern, irritability, change in appetite, concentration difficulty, tiredness or fatigue, *and* loss of sexual interests *for the BDI-SOM-B subscale.

#### 2.2.2. Migration-Related Stressors (MRS)

Different types of MRS were assessed using a list of 25 questions, originally formulated by Lujic (2008) [18] for the treatment evaluation of Turkish migrants in Germany. We adapted this list for our Vietnamese sample by replacing formal relevant aspects (e.g., “*Do you miss your family in Vietnam*” instead of “*Do you miss your family in Turkey*”) but with leaving the main content untouched. One item was deleted because of addressing a topic not relevant for Vietnamese migrants (item number 25: “*Do you have problems at work or with other persons, because of fasting, rejecting pork meat, wearing a headscarf, praying five times a day or washing hands and feet before praying*”). With each of the remaining 24 questions subjects were asked categorically if the particular stressor has occurred (“*yes*” = 1) or did not occur (“*no*” = 0) during the migration process.

Single item scores of the MRS-list were summed up to create an index of stressor-quantity (SQ) with a higher score standing for a larger number of perceived stressors. Scores were calculated when more than 75% of the relevant data were available.

### 2.3. Statistical Analysis

We calculated a dropout-analysis to assess possible differences in* age *(*t*-test)*, gender* (Fisher's exact test), and* level of education *(*chi-squared *test) among participants with less and more than 25% missing MRS-data.

For the main analysis, hierarchical linear regression models were performed to identify the impact of the MRS-quantity on self-reported depressiveness. In the first step, the sociodemographic variables* age*,* gender, *and* level of education* were entered as control variables and in the second analysis step the SQ as the primary predictor. The BDI-II total and subscores, derived from the Buckley et al. (2001) model [20], were included separately as primary outcome measures.

Post hoc analyses were conducted on single item level for those subscales being significantly influenced by the SQ. Therefore, hierarchical linear regression models using the SQ as predictor and the corresponding BDI-II single items as outcome measures were carried out, while all control variables remained unchanged.

Statistical analyses were calculated with IBM SPSS (Version 22). Values of *p* ≤ 0.05 were considered as statistically significant and values of *p* ≤ 0.001 as highly significant. All reported *p* values resulted from two-tailored testing.

## 3. Results

### 3.1. Demographic-, Clinical-, and Migration-Related Sample Characteristics

Table 1 shows that in total 137 Vietnamese outpatients met the inclusion criteria of our study: having at least one depressive episode (F3x.x) according to ICD-10 and having rated more than 75% of the MRS-items. None of them fulfilled the diagnostic criteria for a posttraumatic stress disorder (PTSD). The mean BDI-II total score was 29.96 (SD = 13.58), indicating a moderate to severe symptomatology for this outpatient sample. At the date of the first admission, depressive symptoms persisted for an average time of 23.2 (SD = 25.6) months, reflecting a delayed mental health care utilization.

All subjects were first-generation migrants, that is, born in Vietnam and outside the host country (currently Germany). The mean age of the 26 (19.0%) male patients and 111 (81.0%) female patients at the time of migration was 28.0 (SD = 10.9) years and at the date of the clinical interview mean age was 44.5 (SD = 11.7) years. Although the average duration of years lived in Germany was 17.0 (SD = 10.4), more than 60% of the patients rated their German language skills as “*very poor*” or “*nonexistent*”: a potential confounder that was prevented by translating all self-report instruments into written Vietnamese language. All analyses were controlled for gender and educational status.

On average Vietnamese outpatients reported almost nine (*M* = 8.76; SD = 4.84) stressful and distinct migration-related experiences. The five most frequently reported MRS were as follows: (1) communication problems in Germany (item (1), *n* = 85), (2) longing for the family in Vietnam (item (19), *n* = 71), (3) difficulties in adapting to the German society (item (20), *n* = 63), (4) ambiguity about what to do in certain situations (item (16), *n* = 54), and (5) feeling lonely or isolated in Germany (item (7), *n* = 51). All 24 stressor items are listed in Table 2.

### 3.2. Psychometric Properties of the Applied Instruments

In the present study, the average intercorrelation (Cronbach's *α*) of all 21 BDI-II items was *α* = .94. Regarding the BDI-II subscales, Cronbach's *α* was .74 for the BDI-AFF-B scale, .77 for the BDI-SOM-B scale, and .91 for the BDI-COG-B scale, indicating a moderate to high internal consistency for the respective outcome measures.

Concerning the questionnaire on migration-related stressors, Cronbach's *α* for all 24 MRS-items was .82 demonstrating a sufficiently high internal consistency for the implemented instrument.

### 3.3. Drop-Out Analysis

The data of in total 24 participants were not included in the analysis because here more than 25% of the MRS-information was missing. The response rate for the MRS-questionnaire was 85.1%. We found no significant divergence in age, gender, and level of education between the groups of included and excluded participants (all *p*'s > 0.05).

### 3.4. Regression Analyses: SQ as Predictor and BDI-II Scores as Outcome Measure

The results (Table 3) of the hierarchical linear regression models revealed a substantial effect of the SQ-predictor on the BDI-total score (*F*[4,58] = 3.00, *p* = 0.03^*∗*^, and *η*^2^ = .11), with a higher magnitude of experienced MRS associating with a higher BDI-TOT score.

For the subscales we found a significant effect of the SQ-predictor only for the BDI-COG-B (*F*[4,57] = 4.19, *p* = 0.01^*∗∗*^, and *η*^2^ = .17) subscore but not for the BDI-AFF-B (*p* = 0.13) and BDI-SOM-B subscores (*p* = 0.13). For the cognitive subscale, again a larger number of experienced MRS predicted a higher score on the scale. The sociodemographic factors* age*,* gender, *and* level of education* as control variables had no substantial effect on the results (all *p*'s > 0.05).

### 3.5. Post Hoc Analysis for Those BDI-II Subscales Being Predicted by the SQ

In a further step, we picked out the cognitive subscale for a post hoc analyses on single item level. A set of hierarchical linear regression models was performed again using the SQ as a predictor and the respective BDI-II items as a separate outcome measure. We found for the BDI-COG-B scale the symptoms* pessimism *(*F*[4,57] = 4.07, *p* = 0.01^*∗∗*^, and *η*^2^ = .16),* past failure *(*F*[4,58] = 3.44, *p* = 0.04^*∗*^, and *η*^2^ = .10),* guilt feelings *(*F*[4,57] = 3.94; *p* = 0.01^*∗∗*^; *η*^2^ = .16),* punishment feelings *(*F*[4,57] = 3.44, *p* = 0.01^*∗∗*^, and *η*^2^ = .14), and* suicidal thoughts *(*F*[4,51] = 2.64, *p* = 0.04^*∗*^, and *η*^2^ = .11) being predicted by the SQ, with a higher stressor-quantity leading to a stronger manifestation of the respective symptom. The SQ-predictor did not significantly influence the items* worthlessness* (*p* = 0.16) and* sadness* (*p* = 0.27) of the BDI-COG-B scale.

## 4. Discussion

The overall aim of the study was to investigate how migration-related stressors (MRS) modulate the self-reported severity level and symptom pattern of depression among Vietnamese migrants in Germany. Understanding this mechanism may in turn help to develop migration-sensitive and population-tailored treatment options.

In this respect, a strong association between the number of experienced MRS and the magnitude of psychological distress was found. In detail, more stressors were linked to a higher degree of self-reported depressiveness in general and to a higher extent of the cognitive depression subscale in particular. Within the latter, the symptoms* pessimism*,* past failure*,* guilt feelings*,* punishment feelings, *and* suicidal thoughts* were worsened by an increased count of reported stressors.

### 4.1. The Relation between Migration Stressors and Depression

A correlation between the MRS-quantity and the depression severity is in line with several studies indicating a strong association between stressful migration-related experiences and a broad range of mental health problems across different populations (e.g., [21]). However, in most studies this well-documented association has not been further investigated. One exception is the field of PTSD-research where the so-called “building-block-effect” provides a theoretical framework about how this kind of psychological complaint develops under the experience of traumata [22]. However, since the mechanism underlying a PTSD-disorder is not simply transferable to other psychiatric disorders, studies regarding the question of how nontraumatic but highly stressful migration-related burdens are related to the occurrence and course of affective disorders are still lacking. At the same time, such an examination of how the number of adverse events impacts the mental health status could provide an understanding for developing migration-adapted treatment options.

Through analyzing the different dimensions of depression, we were able to show that the amount of perceived MRS mainly influences the cognitive (and less the affective or somatic) symptoms of depression. This impact seems to follow a dose-response relation with a higher count of experienced stressors leading to a greater extent of the cognitive depression symptoms in the BDI-II, especially regarding the items* pessimism*,* past failure*,* guilt feelings*,* punishment feelings, *and* suicidal thoughts.* This finding is in line with a suggestion of Bhugra and Ayonrinde (2004) to pay particular attention to migration-associated dysfunctional cognitions when dealing with depressed migrants and refugees [23]. Simultaneously, these authors state that extensive research is needed to investigate further the kind of dysfunctional cognitive schemata prone migrants and refugees (even more) vulnerable for an affective disorder.

### 4.2. MRS and the Cognitive Depression-Dimension: An Explanatory Approach

In most cases, the dynamic process of migration does not entail exclusively either advantages or disadvantages but often a mixture of both. On the one hand, migration can have a positive effect on mental health, for example, by inducing optimism and hope as well as by leading to an actual improvement of the current life situation [24]. However, moving to another country does not always result in positively perceived changes. Many immigrants are confronted by an array of obstacles, such as perceived discrimination, the absence of social networks, homesickness, lack of feelings of belonging, sense of guilt for leaving family behind, language barriers, and an uncertain resident status [25]. Considering these two possible trajectories of the migration process at one point or another a more or less attentive, intrapsychic weighing up of the so far experienced migration benefits and costs is conceivable, causing depressogenic thoughts and feelings if the result happens to be negative.

With regard to our results such a comparison could have the following implications: (1) the less advantageous this psychological evaluation process turns out, the more intense the perception of failure, hopelessness, and resignation may be. This finding could explain the dose-effect, with more reported MRS leading to a higher self-reported depression-outcome. (2) Since such a comparison requires cognitive efforts, a negative result, in turn, could play a part when forming the cognitive dimension of depression. This may explain our finding that primarily cognitive symptoms of depression were related to the MRS-quantity. However, at the same time, it is important to bear in mind that there is a growing body of research documenting the substantial contribution of emotions and affects in the way a migrant experiences his or her own identity and fate [26]. Therefore, we do not claim the proceeding evaluation process to be exclusively a cognitive one, without taking into account any emotional states or affective perception, which are essential for any cognitive processing. (3) An undesirable outcome could finally result in an overall pessimistic perception of the past (in our study the BDI-II symptoms* past failure*,* guilt feelings,* and to some extent* punishment feelings*) and future (in our study the BDI-II symptom* pessimism*). Such a global negative perception may, in turn, consolidate the belief that no matter what and how hard the person is trying to improve the situation the results of his or her efforts turn out to be negative. This experience of uncontrollability and heteronomy may be similar to a mindset commonly referred to as* learned helplessness *[27]. This psychological model declares the conviction of having no influence over one's destiny to be a major risk factor for the development of symptoms of depression. Therefore future studies should also account for a migrant's attribution style and locus of control when further investigating associations between MRS and depression.

However, regarding this psychological evaluation process, it is important to keep in mind that the weighing up of migration benefits and drawbacks most likely is not a one-time action. It may be rather a dynamic process being repeated several times during migration and its different stages [28]. Accordingly, studies have shown that while the mental health status of newly arrived immigrants often exceeds the one of the host population (being referred to as the “healthy-migrant-effect”; see [29]), this advantage vanishes over time, and the immigrants' mental health status resembles that of the residents. For the present sample, this healthy-migrant-effect may play a subordinate role since the average time spent in Germany was at admission seventeen years. Nevertheless, regarding future studies, it might be beneficial to apply a longitudinal design to understand how the mental health condition of Vietnamese migrants develops during their stay in Germany (see also Section 4.5.: Implications for Clinical Practitioners and Future Research).

### 4.3. Migration Stressors and Suicidal Ideation

Particular attention should be placed on our finding of an increase in suicidal thoughts under the influence of MRS. This elevation of suicidality is consistent with current studies on other migrant populations. For instance, Latino immigrants in Spain and the United States have revealed a rise in suicidal ideation in the light of discrimination and a weak sense of belonging [30]. Also among African American college students an elevated risk for suicidal thoughts was reported, especially when they experienced high acculturative stress or a poor group identity [31]. The same augmentation of suicidality in the context of migration problems has been reported for Polish and Turkish migrants being compared to a German control sample [32] as well as for American Indians in comparison to the US host population [33].

For Asian migrants, this rise in suicidal ideation under MRS may be further aggravated by their tendency to present somatic instead of actual existing psychological complaints (including suicidal ideation) when approaching primary health care [34]. This renders the detection of suicidality among this migrant population challenging and underscores the supplementary use of risk profiles, such as the report of a large amount of experienced MRS, when performing clinical clarification.

### 4.4. Limitations and Strengths

This study has several limitations that have to be addressed:

(1) Due to the relatively small sample size and the constraint on Vietnamese psychiatric outpatients in Berlin seeking help voluntarily our results are not generalizable to other migrant population without caution.

(2) Since there is no standardized tool for assessing MRS in migrants from Southeast Asia, we used our questionnaire in a more explorative way covering a broad as possible range of MRS.

(3) Additionally, assessment of primary outcomes was limited to self-rated depressiveness and perceived MRS. However, self-report questionnaires may encourage respondents from sociocentric cultures as Vietnam to answer less in consideration of social-desirability because of perceived anonymity.

(4) Although we performed a dropout-analysis on patients with more than 25% missing MRS-data and found no significant deviations in age, gender, and level of education, a sampling bias cannot be fully excluded.

(5) Finally, due to the cross-sectional design of our study limiting causal attribution, we could not fully exclude the possibility that a higher level of depressiveness is not only the result but also the reason for more reported MRS [35]. Therefore, future research should apply a longitudinal design including a healthy (Vietnamese) control group to clarify the pathway between experienced MRS and depression further.

Despite these limitations, the present study is one of the very few on the mental health status of Vietnamese migrants in Germany and the first to examine an association between experienced migration-related stressors and the occurrence and severity of depression among this population. Our findings thus may be seen as a promising starting point for future investigations as well as for the development of culture-sensible therapy options to enhance the treatment adherence.

### 4.5. Implications for Clinical Practitioners and Future Research

Considering the delayed and diminished mental health care utilization of Vietnamese migrants our study provides the following recommendations for clinical practitioners: (1) The diagnostic assessment of Vietnamese psychiatric patients should not exclusively focus on traumatic but also on non-life-threatening but highly stressful migration-related experiences. (2) Therapeutic interventions for depressed (Vietnamese) migrants should pay attention to distorted beliefs and cognitions about recurring migration-related stressors. This assumption is in line with studies on Vietnamese patients with PTSD, treated with a culturally adapted version of cognitive-behavioral therapy [36, 37]. Although the mechanisms underlying a PTSD and a MRS-related mood disorder may differ, in both cases the common experience of not having sufficient coping capacity to handle stressors may play a major role. Such a lack of skills or coping strategies may leave the affected person more vulnerable to self-destructive thoughts and depression-related cognitions. (3) Furthermore, a supplemental screening for suicidal tendencies should be imposed for (Vietnamese) patients reporting a broad range of exposed migration stressors.

Since our study was primarily concerned with the negative aspects of the migration process, future investigations should also consider its benefits, such as an improved socioeconomic status or the access to a better health care or educational system [24]. Thus, regarding a psychological evaluation process comparing improvements and deteriorations, it is important to keep in mind that migration can influence mental health in both ways, negative and/or positive.

Therefore, future research should apply a longitudinal design, to further investigate how the mental health condition of Vietnamese migrants in Germany varies with the length of their stay in the host country. Also, a mixed-method-approach, combining quantitative and qualitative data, is conceivable for understanding the cognitive and emotional disunity when migrants are confronted with diverging cultural and social requirements and ambivalences. Individual and extended case studies would complete the picture by providing further qualitative information on when, why, and how often a migrant takes stock of his or her migration process.

## Figures and Tables

**Table 1:** Sample characteristics (*N* = 137).

	*n* ^a^ (%)	*M*(SD)^b^
*Sociodemographics*		
Gender		
Male	26 (19.0%)	
Female	111 (81.0%)	
Age (in years)		44.5 (11.7)
Status of education		
No education	75 (58.1%)	
Education completed	36 (27.9%)	
Studies completed	18 (14.0%)	
Working status		
In work	32 (24.5%)	
Unemployed	71 (54.2%)	
Retired	7 (5.3%)	
No working permission	21 (16.0%)	
German language skills		
C1/B2	21 (15.8%)	
A2/B1	31 (23.3%)	
A1/none	81 (60.9%)	
*Acculturation characteristics*		
Age at migration		28.0 (10.9)
Years lived in Germany		17.0 (10.4)
Reason for migration		
Marriage migration	8 (6.3%)	
Family reunification	22 (17.4%)	
Labor migration	72 (57.1%)	
Educational reason	5 (4.0%)	
Asylum seeker	12 (9.5%)	
Other reason	7 (5.7%)	
Residential status		
Secure	117 (86.0%)	
Unsecure	19 (14.0%)	
*Clinical characteristics*		
ICD-10 main diagnosis		
F32.0	8 (5.8%)	
F32.1	43 (31.4%)	
F32.2	7 (5.1%)	
F33.0	8 (5.8%)	
F33.1	40 (29.2%)	
F33.2	17 (12.4%)	
Other F3-diagnosis	14 (10.3%)	
Average length of symptomatology (in months)		23.2 (25.6)
At least one psychiatric comorbidity^c^	65 (47.5%)	

^a^Subsample (values vary because of missing data). ^b^Mean (standard deviation). ^c^Psychiatric comorbidity was assessed with the M.I.N.I. 5.0 and refers to ICD-10.

**Table 2:** Prevalence rates for the 24 migration-related stressor items (adapted from Lujic 2008).

Stressor	*M* ^a^	SD^b^	FRQ_*r*_^c^	FRQ_*a*_^d^	%^e^
(1) Do you have communication problems because of insufficient German language skills?	0.88	0.33	97	85	87.6
(2) As a child, did your reference persons change often because of migration?	0.16	0.37	93	16	17.2
(3) As a child, were you separated from your parents over a longer period of time because of migration?	0.23	0.42	91	20	22.0
(4) Are your children still in Vietnam?	0.28	0.45	90	27	30.0
(5) Have your parents passed away, while you have been in Germany?	0.32	0.47	94	44	46.8
(6) Did your children have conflicts with the police or law in Germany?	0.03	0.18	89	3	3.4
(7) Do you feel lonely or socially isolated in Germany?	0.51	0.50	96	51	53.1
(8) Were you excluded from your original family?	0.40	0.50	89	34	38.2
(9) Was your partner/husband rejected from your original family?	0.14	0.35	84	11	13.1
(10) Do you feel politically disadvantaged in Germany?	0.26	0.44	91	24	26.4
(11) Do you feel religiously disadvantaged in Germany?	0.07	0.26	92	9	9.8
(12) Do you feel like a stranger in the German society?	0.42	0.50	96	44	45.8
(13) Were you discriminated in Germany because of origin?	0.23	0.43	95	25	26.3
(14) Do you feel separated or isolated from your family because of different views of life?	0.21	0.41	85	21	24.7
(15) Do you feel separated or isolated from other relevant persons because of different views of life?	0.28	0.45	89	31	34.8
(16) In certain situations, do you feel unsure about what to do or what is expected from you?	0.58	0.50	90	54	60.0
(17) Do you have marital problems because of differing views on task-distribution?	0.33	0.48	84	30	35.7
(18) Do you wish to return to your home country?	0.40	0.50	86	30	34.9
(19) Do you miss your family in Vietnam?	0.79	0.41	93	71	76.3
(20) Do you experience difficulties in adapting to the German society?	0.68	0.47	90	63	70.0
(21) Do you have the impression that many of your hopes or expectations did not come true in Germany?	0.49	0.50	89	43	48.3
(22) Do you wish on the one hand to return to your home country and on the other hand to stay in Germany?	0.53	0.50	90	48	53.3
(23) Have you already experienced the Germans as being hostile?	0.19	0.40	89	16	18.0
(24) Do you sometimes feel pressured by the Vietnamese community because of having divergent beliefs or expectations?	0.21	0.41	91	20	22.0

^a^ *M = *mean; ^b^SD = standard deviation;  ^c^FRQ_*r*_* = *frequency (rated): number of times the item was rated; ^d^FRQ_*a*_ = frequency (affirmed): number of times the item was affirmed; % = percentage of affirmed items in relation to the number of rated items (FRQ_*a*_*∗*100/FRQ_*r*_).

**Table 3:** Hierarchical regressions^a^ of the MRS-quantity on different facets of self-reported depressiveness (BDI-II).

Outcome	*M*(SD)^b^	*B* ^c^	${R^{2}}^{\overset{\;}{d}}$	*β* ^e^	CI (95%)^f^	*F* ^g^	*p* ^h^
*BDI_TOTAL *	29.96 (13.58)	1.12	0.17	0.41	0.43–1.80	3.00	0.026^*∗*^
Buckley et al. (2001)							
BDI-AFF-B	5.93 (2.88)	0.19	0.12	0.33	0.04–0.35	1.86	0.13 n.s.
*BDI-COG-B*	11.13 (6.50)	0.59	0.23	0.46	0.28–0.89	4.19	0.005^*∗∗*^
BDI-SOM-B	13.05 (5.57)	0.33	0.11	0.30	0.05–0.26	1.84	0.13 n.s.

^a^Control variables: *age, sex, and level of education.  *^b^Mean (standard deviation). ^c^Unstandardized beta-coefficient. ^d^Coefficient of determination. ^e^Standardized beta-coefficient. ^f^Confidence interval. ^g^*F*-value. ^h^Level of significance: ^*∗*^*p* ≤ 0.05; ^*∗∗*^*p* ≤ 0.01;  n.s. = not significant.

## Additional Points

*Why This Study?* Little is known about the mental health condition of Vietnamese migrants (in Germany) and how acculturative stressors influence their perception of depressive symptoms. This is the first study to investigate if and how the quantity of experienced migration-related stressors influences different facets of self-reported depressiveness (i.e., the overall severity level as well as the affective, cognitive, and somatic dimension of depression) in a sample of clinically depressed Vietnamese migrants in Germany.* What This Study Has Revealed?* A dose-response-effect was found, with a higher number of experienced migration-related stressors being associated with higher self-reported severity level of depression in general as well as a greater level of cognitive depression symptoms (i.e., pessimism, past failure, guilt feelings, punishment feelings, and suicidal thoughts) in particular.* What Are the Practical Implications?* For the future development of culturally adapted and person-centered Depression treatment programs, our findings suggest placing a stronger focus on migration-related stressors with particular attention to dysfunctional beliefs and cognitions (including suicidal thoughts) as a result of perceived stressful migration experiences.
